# *Cleistanthus collinus* induces type I distal renal tubular acidosis and type II respiratory failure in rats

**DOI:** 10.4103/0253-7613.66843

**Published:** 2010-06

**Authors:** Delinda Maneksh, Anita Sidharthan, Kavithapriya Kettimuthu, Praghalathan Kanthakumar, Amala A. Lourthuraj, Anup Ramachandran, Sathya Subramani

**Affiliations:** Department of Physiology, Dr. Somervell Memorial C.S.I. Medical College and Hospital, Karakonam, Kerala, India; 1 Department of Physiology, Christian Medical College, Vellore, Tamilnadu, India; 2 Wellcome Research Laboratory, Christian Medical College, Vellore, Tamilnadu, India

**Keywords:** *Cleistanthus collinus*, distal renal tubular acidosis, proton pump, suicide

## Abstract

**Background and Purpose:**

A water decoction of the poisonous shrub *Cleistanthus collinus* is used for suicidal purposes. The mortality rate is 28%. The clinical profile includes distal renal tubular acidosis (DRTA) and respiratory failure. The mechanism of toxicity is unclear.

**Objectives:**

To demonstrate features of *C. collinus* toxicity in a rat model and to identify its mechanism(s) of action.

**Materials and Methods:**

Rats were anesthetized and the carotid artery was cannulated. Electrocardiogram and respiratory movements were recorded. Either aqueous extract of *C. collinus* or control solution was administered intraperitoneally. Serial measurements of blood gases, electrolytes and urinary pH were made. Isolated brush border and basolateral membranes from rat kidney were incubated with *C. collinus* extract and reduction in ATPase activity was assessed. Venous blood samples from human volunteers and rats were incubated with an acetone extract of *C. collinus* and plasma potassium was estimated as an assay for sodium–potassium pump activity.

**Results:**

The mortality was 100% in tests and 17% in controls. Terminal event in test animals was respiratory arrest. Controls had metabolic acidosis, respiratory compensation acidic urine and hyperkalemia. Test animals showed respiratory acidosis, alkaline urine and low blood potassium as compared to controls. *C. collinus* extract inhibited ATPase activity in rat kidney. Plasma K^+^ did not increase in human blood incubated with *C. collinus* extract.

**Conclusions and Implications:**

Active principles of *C. collinus* inhibit proton pumps in the renal brush border, resulting in type I DRTA in rats. There is no inhibition of sodium–potassium pump activity. Test animals develop respiratory acidosis, and the immediate cause of death is respiratory arrest.

## Introduction

*Cleistanthus collinus* is a highly poisonous shrub, which belongs to the family Euphorbiaceae. A water decoction of the leaves is used for suicidal purposes in many parts of southern India. The victim is brought to the hospital in a conscious state and then develops complications such as metabolic acidosis, hypokalemia, hypotension, cardiac arrhythmias, muscular weakness and respiratory and renal failure.[1–4] The mortality is 28%, and death usually occurs 3–7 days after ingestion of the poison.[1] The mechanism of toxicity is unclear. Treatment is symptomatic, involving bicarbonate and potassium supplementation, temporary cardiac pacing and mechanical ventilation if required.

Case reports of two patients who died of *C. collinus* poisoning show that they developed hypokalemia, hypotension, cardiac arrhythmias, mixed metabolic and respiratory acidosis and renal failure.[2] High urinary pH was the other finding. The triad of metabolic acidosis, hypokalemia and relatively alkaline urine occurs specifically in type I or type II renal tubular acidosis (RTA).[5] Benjamin *et al*. showed that a patient with *C. collinus* poisoning had cardiac arrhythmia, hypoxia despite oxygen supplementation, hypokalemic metabolic acidosis and urinary pH of 6.5.[3] They clearly recognized type I distal RTA (DRTA) along with ARDS and distributive shock. This patient was successfully treated with N-acetylcysteine. The rationale for using N-acetylcysteine was that cysteine has been shown to reduce the mortality in *C. collinus*-poisoned rats.[6] Common findings in all these clinical studies are hypokalemia, metabolic acidosis, respiratory failure, hypotension and cardiac arrhythmias.[1–4]

In addition to these clinical findings, experiments using animal models have proposed various mechanisms of action for *C. collinus*, including inhibition of sodium–potassium pump, inhibition of LDH isoenzymes, depletion of glutathione and neuromuscular blockade causing respiratory paralysis.[7–9]

Cleistanthin A and B are shown to hamper cell growth and proliferation by interfering with the replication and transcriptional processes. Cleistanthin A has also been shown to have significant anticancer activity, and causes tumor regression in mice by inducing apoptosis.[10,11] Antioxidants like cysteine and melatonin may help in reducing the brain damage caused by the toxin. However, these substances do not prolong the survival time in rats poisoned with the *C. collinus* toxin.[6,12]

The aim of this study was to develop an animal model of toxicity and to identify the molecular mechanism(s) of action of the toxin.

## Materials and Methods

### Aqueous extract of *C. collinus*

Thirty-five grams of powdered dried leaves were taken in 350 ml distilled water, heated in a boiling water bath for 10 min, filtered and concentrated at 70°C to a volume of 50 ml. Osmolarity of the extracts used in the test rats was 500–600 mosm/L, pH was 4–5 and potassium concentration 50–100 mM.

### Control solution

Control solutions were made to match the aqueous extract in terms of potassium, pH and osmolarity, for use in control rats.

### Acetone extract of *C. collinus*

Dry *C. collinus* leaves were delipidated with hexane and extracted with acetone. The extract was filtered, concentrated and dried. The acetone extract was prepared for *in vitro* blood experiments because the aqueous extract was rich in potassium and the assay was determination of K^+^ concentration in blood.

### Rat in vivo experiments

Male or female Wistar rats (150–290 g) were used. Experimental methods were approved by the Institutional Animal Ethics Committee. Rats were anesthetized with either thiopentone (40 mg/kg) or ketamine (75–100 mg/kg) given intraperitoneally. Electrocardiogram (ECG) was recorded with subcutaneous electrodes. Respiration was monitored with a force transducer hooked to the ventral abdominal wall. The carotid artery was cannulated and the animal was heparinized with 200 IU heparin. 0.2 ml arterial blood was drawn at different intervals after administration of the toxin or control solution and serial measurements of whole blood pH, bicarbonate, potassium, PCO_2_ and PO_2_ were made using an automated analyzer. Urine was collected as it formed and urine pH was measured.

Onset of experiment (0 h) was taken as the time of drawing the first arterial blood sample following carotid cannulation. Within 5–15 min after carotid cannulation, the toxic extract or control solution was administered intraperitoneally. The test animals were continuously monitored till death and the control animals were monitored for 6–8 h, after which they were sacrificed if death had not occurred. Death was assessed by cessation of respiration and ECG complexes.

Some animals tested before dose standardization received *C. collinus* extracts with osmolarity as low as 200 mosm/L and as high as 800 mosm/L. These test animals were included in the overall mortality assessment. The data from these animals were also included for urine pH comparisons. As for the blood gas and electrolyte measurements, test rats (*n* = 7) that received the aqueous extract of *C. collinus* with osmolarity of 500–600 mosm/L (0.25–0.6 ml) alone were considered for analysis. Control rats (*n* = 12) received a similar quantity of the control solution. Only those animals that had a starting pH of 7.25–7.35, PCO_2_ of 35–55 mmHg and PO_2_ of 75–120 mmHg were included in the analysis.

### Preparation of renal brush border membrane (BBM) and basolateral membrane (BLM) and ATPase assay

Rats were killed by cervical dislocation, the kidneys were removed and homogenized and renal BBM was prepared as described by Basivireddy and Balasubramanian.[13] Purity of BBM was checked by enrichment of alkaline phosphatase (ALP). Renal BLM was isolated as described by Pritchard and Renfro (1983) with slight modifications and purity was checked by enrichment of total ATPase activity.[14] Different aliquots of the isolated membrane preparations (500 *µ*g of protein) were incubated with aqueous extracts of *C. collinus* (50 µl of 230 msom/L extract), 1 mM N-ethyl maleimide, its vehicle dimethyl sulphoxide (DMSO) or 10 *µ*M ouabain for 30 min, following which total ATPase activity was measured as described by Quigley and Gotterer.[15] Enzymatic activity was measured by the amount of inorganic phosphate (Pi) released from ATP.

### Red blood cell experiments

The rationale for these experiments was to determine whether *C. collinus* was a sodium–potassium pump blocker, given that it is a glycoside and produces hypokalemia. Venous blood (1 ml) from human volunteers or rat blood (from direct cardiac puncture) were anticoagulated with acid-citrate-dextrose and incubated with the dried acetone extract of *C. collinus* dissolved in acetone, at a final concentration of 0.5 or 1 mg dried extract/ml blood. The dose was chosen based on the dose of the acetone extract of *C. collinus*, which showed 100% mortality in rats (data not shown). Negative controls with acetone and positive controls with ouabain (10 *µ*M), a known sodium–potassium pump blocker, were also run. Care was taken to avoid hemolysis and, if any hemolysis was observed, the experiment was not included in the analysis. The control 4-h plasma potassium concentration was taken as 100% and the percentage change in others was plotted as a category plot.

### Statistical methods

Wilcoxon’s signed rank (WSR) test was used to compare values within the same group at different time points. Mann Whitney U (MWU) test was used for comparison between tests and controls. *P* values < 0.05 was considered statistically significant.

## Results

**Figure 1:**
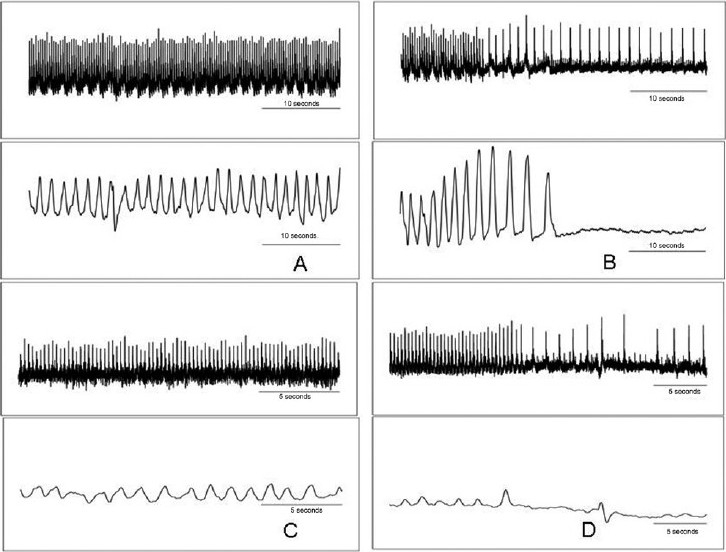
Raw tracings of electrocardiogram (upper panels) and respiratory movements (lower panels) in two animals that received the aqueous extract of *Cleistanthus collinus*. A and B are initial and terminal recordings from one animal and C and D are findings from the other. The terminal event in both animals, as seen in B and D, is respiratory arrest accompanied by a sudden drop in heart rate. The same picture was seen in all test animals.

**Figure 2:**
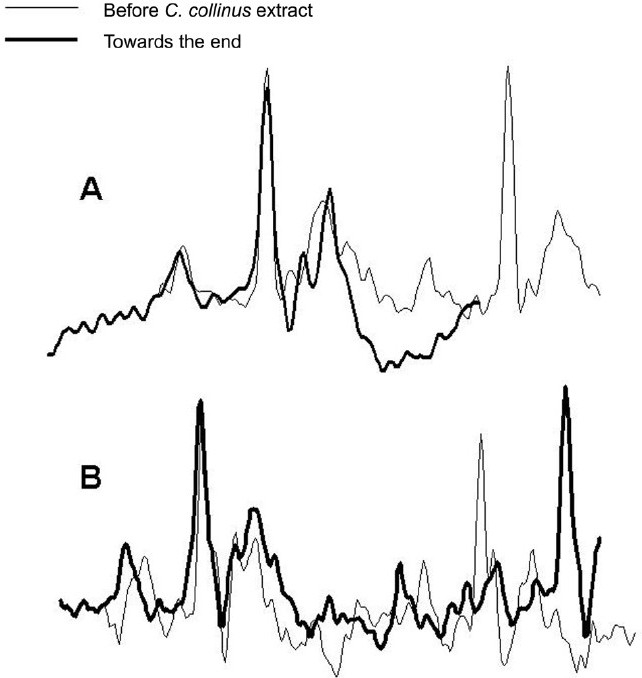
Raw electrocardiogram (ECG) tracings from two test animals. The thin line shows two ECG complexes recorded before the administration of the aqueous extract of *Cleistanthus collinus*. The thick lines represent ECG tracings just prior to death after the administration of the aqueous extract of *C. collinus*. The time interval of both the traces is matched and the first R-wave is superimposed. The upper panel shows no observable change in the ECG pattern before and after *C. collinus* extract (A). The lower panel taken from a different animal shows PR prolongation and increase in T-wave duration (B).

Mortality was 100% with administration of the aqueous extract of *C. collinus*. In 19 test animals given *C. collinus* extract at osmolarities ranging from 200 to 800 mosm/L (0.25–1.0 ml), death occurred in 20 min to 8 h. Of the 12 control rats that received an equivalent volume of control solution, two died and the rest lived for 8 h, after which they were sacrificed (16.6% mortality). Death was assessed by cessation of respiratory movements and ECG complexes. In all cases of tests, the terminal event was abrupt cessation of respiratory movement. Heart rate showed a gradual decrease in all test animals over time. However, a similar degree of bradycardia also occurred in controls and therefore may not be due to the effect of the toxin. A sudden drop in heart rate was observed, coinciding with the respiratory arrest [Figure 1B and D]. The ECG complexes appeared normal even at the end and were seen for many minutes after cessation of respiration. There was either no difference in the ECG complexes at the start and after respiratory arrest [Figure 2A] or there was prolongation of the PR interval [Figure 2B] and, sometimes, the duration of the T wave was higher [Figure 2B].

In the period preceding the respiratory arrest, respiratory movements appeared smaller and there were instances of periodic breathing. In some animals, increased chest movements were seen just before the respiratory arrest [Figure 1B]. In most of the control animals, there was hyperventilation during the course of the experiment, either due to an increase in tidal volume or an increase in respiratory rate.

**Figure 3:**
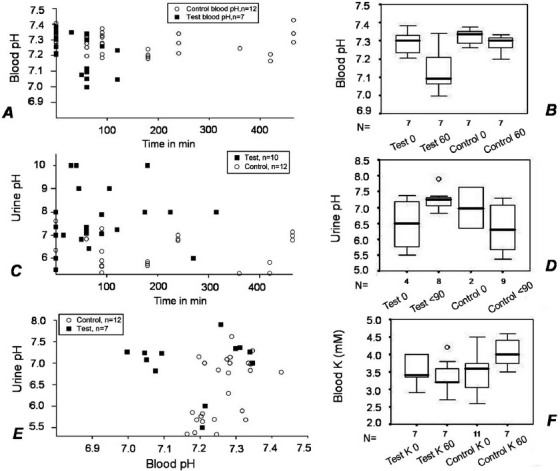
(A and B) After 60 min, acidosis is severe in the test group (*P* = 0.031, WSR test, *n* = 7) than in the control group (*P* = 0.016, WSR test, *n* = 7). (C and D) Urine pH is acidic in controls by 90 min, whereas it remains alkaline in tests (*P* = 0.026, MWU test, n = 6 for test and 7 for control). (E) Urine pH remains high even when blood pH has dropped below 7.1 in tests. (F) Blood potassium has increased significantly in controls (*P* = 0.039, WSR test, *n* = 8) while it has not changed in tests (*P* = 0.297, WSR test, *n* = 7). At 60 min, blood K+ levels in control groups were significantly higher than in tests (*P* = 0.017, MWU test).

*Blood pH*: Acidosis was seen in the control as well as the *C. collinus*-treated groups. The acidosis in test rats was more severe than in controls [Figure 3A and B]. When blood pH values before injection of toxin (0 min) and at 60 min were compared, there was a significant decrease in blood pH in the test rats (*P* = 0.031) as well as in the control rats (*P* = 0.016).

*Urine pH*: It was increased in test animals, whereas it tended to decrease in controls with time [Figure 3C]. Urine pH values within the first 90 min [Figure 3D] were 7.24 ± 0.31 in the tests and 6.36 ± 0.74 in the controls (*P* = 0.026). While urine generally became acidic when blood pH dropped below 7.2 in controls, urine pH remained alkaline in tests, even when the blood pH dropped below 7.1 [Figure 3E].

### Blood K^+^

There was a significant increase in blood K^+^ in the control groups when the 0 and 60 min values were compared (*P* = 0.039) [Figure 3F]. In the test group, in spite of the severe acidosis, there was no significant change in blood K^+^ between the 0 and 60 min levels (*P* = 0.297). However, the blood K^+^ levels at 60 min in the test group were significantly lower than the corresponding values in the control group (*P* = 0.017). Therefore, although frank hypokalemia was not seen, blood K^+^ was lower in the test group as compared to the control group.

### Blood PCO_2_

**Figure 4:**
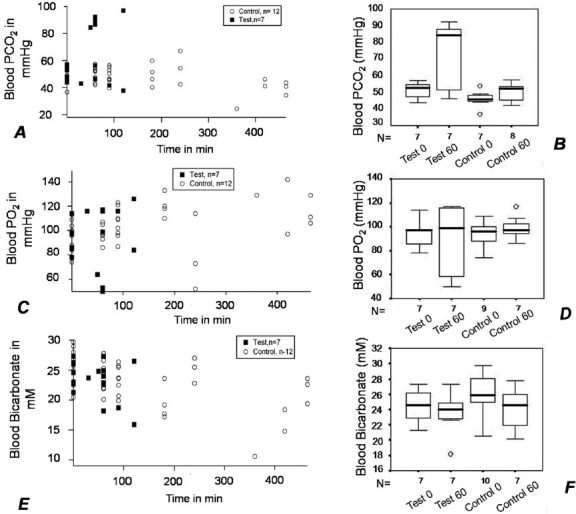
(A and B) Blood PCO2 increases in tests by 60 min (*P* = 0.047 with WSR test, *n* =7) while it remains unchanged in controls (*P* = 0.219 with WSR test, *n* = 7). (C and D) Blood PO2 at 60 min shows a wide variability in tests and, therefore, no significant change was observed as compared to the initial values (*P* = 0.609 with WSR test, *n* = 7). In controls, PO2 remained unchanged (*P* =1 with WSR test, *n* = 6) at 60 min. (E and F) Blood bicarbonate remained unchanged in tests (*P* =0.578, WSR test, n = 7) and controls (*P* =0.156, WSR test, n=7) at 60 min as compared to the initial values.

While there was no significant difference in controls between the pre- and post-injection values at 0 and 60 min (*P* = 0.219), there was a significant increase in the blood PCO_2_ values in tests at 60 min (*P* = 0.047) [Figure 4A and B].

### Blood PO_2_

There was no significant change in the PO_2_ values in the controls between 0 and 60 min (*P* = 1; Figure 4C and D). Although there was wide variability in the values at 60 min in the tests, as is evident in the box plot [Figure 4D], there was no statistically significant change in the PO_2_ values in the tests also at 60 min (*P* = 0.609).

### Blood bicarbonate

The blood bicarbonate levels did not change significantly over the first hour in the test and control rats [Figure 4E and F]. There was no statistically significant difference between pre- and post-intervention values at 60 min in the controls (Figure 4F; *P* = 0.156) and in the tests (*P* = 0.578). However, there was a significant decline in bicarbonate values in the control group toward the later stages (400 min and above) when compared with the 0-min values (*P* = 0.031 by WSR test, n=6).

### Renal ATPase assay

**Figure 5:**
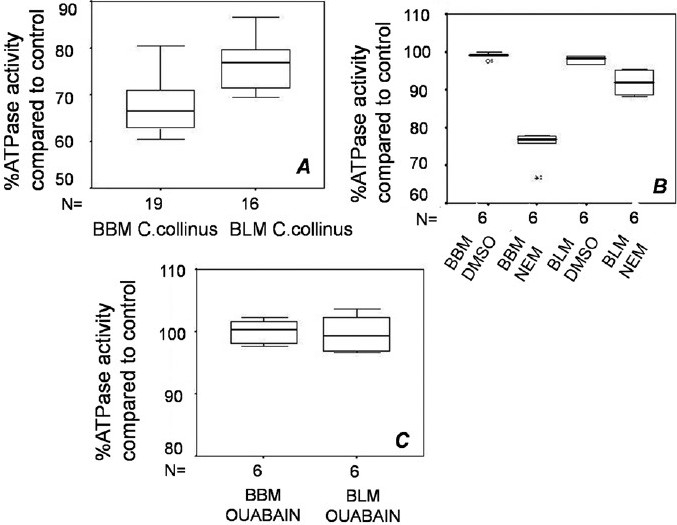
(A) Incubation with *Cleistanthus collinus* extract decreases ATPase activity of both brush border (BBM) and basolateral membranes (BLM) of rat kidney (*n* = 4, *P* < 0.001 with WSR test in both cases). (B) N-ethyl maleimide (NEM), a known proton pump blocker, also inhibited ATPase activity of both BBM and BLM as compared to the vehicle DMSO, in which it was dissolved (*n* = 4, *P* = 0.03 with WSR test). (C) Ouabain, a known sodium–potassium pump blocker, did not inhibit the ATPase activities of both BBM and BLM (*P* = 1 for BLM, *n* = 6 and *P* = 0.2 for BBM, *n* = 9).

Total ATPase activities of the isolated BBM and BLM of rat kidney were inhibited when treated with the *C. collinus* extract (Figure 5A; *P* < 0.001). N-ethylmaleimide (NEM), a known proton pump blocker, also showed significant reduction in the ATPase activity of both membrane fractions (Figure 5B; *P* = 0.031) as compared to the vehicle DMSO. Ouabain, a known sodium–potassium pump blocker, failed to show any inhibition of ATPase activity of both membranes (Figure 5C; *P* = 1 and *P* = 0.2).

### sodium–potassium ATPase activity

**Figure 6:**
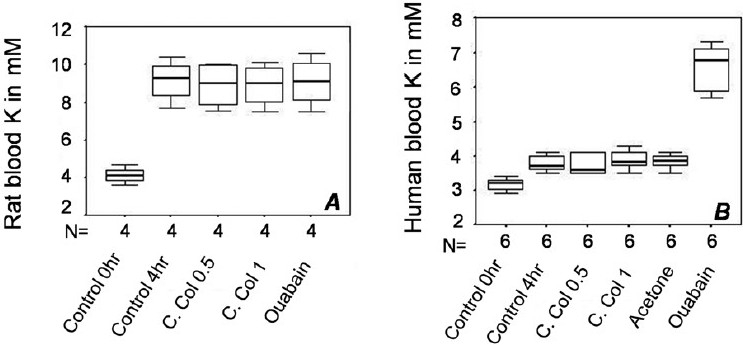
(A) In rat blood samples incubated with or without additives, there was an increase in plasma K^+^ after 4 h. There was no difference between untreated controls after 4 h and samples treated with either acetone extract of *Cleistanthus collinus* (*n* = 4, *P* = 0.625, with WSR test for both doses of *C. collinus*) or Ouabain (*n* = 4, *P* = 1 with WSR test). (B) In human blood, while ouabain caused a significant elevation of blood K+ after 4 h (*n* = 6, *P* = 0.031 with WSR test), there was no change in samples incubated with *C. collinus* extract (*n* = 6, *P* = 0.25 with WSR test).

In rat blood, there was a doubling of plasma K^+^ even in negative controls, which did not have any additive [Figure 6A]. However, the values of plasma K^+^ for the *C. collinus* (in doses of 0.5 mg/ml and 1 mg/ml blood) and the ouabain (10 *µ*M) groups incubated for 4 h were not different from the control blood incubated for 4 h (*P* = 0.625 for both doses of *C. collinus* and *P* = 1 for ouabain). In case of human blood [Figure 6B], there was no increase in plasma K^+^ in samples incubated with both doses of the *C. collinus* acetone extract (*P* = 0.25), while an increase was seen with ouabain (*P* = 0.031).

## Discussion

Rat *in vivo* experiments showed a clear difference between controls and tests. In controls, there was significant acidosis by 1 h, probably due to the surgical procedures involved. Although blood bicarbonate was not significantly lower in these animals by 1 h, the levels had dropped significantly in the later hours, suggesting that the acidosis in controls is metabolic. Decrease in urine pH and increase in blood potassium were compatible with usual responses to metabolic acidosis other than type I and II RTAs. Although, initially, it seemed that the metabolic acidosis that the control rats develop may be a major limitation to the study, it actually turned out to be a good control for the different type of acidosis that the test rats developed.

In the test group, there was severe respiratory acidosis. Metabolic acidosis was not demonstrable during the short period that the animals lived. Urine remained alkaline in spite of the severe acidosis, indicating failure of the acid-secreting mechanism in the distal renal tubule. Therefore, although bicarbonate levels had not changed significantly by 1 h (when the values were compared), there is a definite indication of a distal RTA type I. Serum potassium levels were lower in test animals than in control animals in spite of more severe acidosis. Lack of a hyperkalemic response to acidosis corroborates the picture of type I DRTA. Type II RTA can also lead to a similar clinical picture; however, it is characterized by HCO3^-^ wastage in urine, which would have led to a drop in blood bicarbonate levels. Because no significant reduction had occurred in blood bicarbonate even while the urine was alkaline, it is concluded that *C. collinus* leads to type I DRTA in rats.

The molecular mechanism of type I DRTA could be inhibition of proton pumps (H^+^ ATPase or H^+^/K^+^ ATPase) on the BBM or inhibition of anion exchanger on the BLM of distal renal tubules. Inhibition of proton pumps was chosen as the first hypothesis to be tested. The *in vitro* experiments on rat kidney demonstrate that there is ATPase inhibition in BBM as well as BLM. N-ethyl maleimide, a known proton pump blocker,[16] also inhibits ATPase activity of both membranes akin to *C. collinus* and serves as a positive control. While BBM has proton pumps only to account for ATPase activity, BLM is rich in sodium–potassium pump, in addition to proton pumps expressed in the beta-intercalated cells.[17] Therefore, it had to be ensured whether the inhibition of ATPase activity of BLM by *C. collinus* extract is due to inhibition of proton pumps in BLM or due to inhibition of Na^+^–K^+^ pumps. The strategy was to test another tissue that expresses sodium–potassium pumps rather exclusively. Estimation of plasma K^+^ after incubation of blood samples with the toxin was a functional assay for the sodium–potassium pump present on erythrocyte membranes. In human blood samples, the *C. collinus* extract did not increase the plasma K^+^ levels after 4 h of incubation, while ouabain, a known blocker of the sodium–potassium pump, did so. Ouabain served as a good positive control for sodium–potassium pump inhibition in human blood. It was, therefore, clear that *C. collinus* extract does not inhibit the human isoform of Na^+^–K^+^ pump. When the same experiment was performed with rat blood, control samples themselves showed doubling of plasma K^+^ after 4 h. There was no further increase with *C. collinus* extract, ruling out sodium–potassium pump inhibition by *C. collinus* in rat blood also. Ouabain did not serve as a positive control for Na^+^–K^+^ pump inhibition in rat blood because there was no increase in plasma K^+^ in rat blood sample incubated with ouabain in comparison to control after 4 h. Lack of effect of ouabain in rat blood is understandable given the ouabain-resistance of certain isoforms of the sodium–potassium pump.[18,19] Incubation of BBM and BLM of rat kidney with ouabain also showed that it did not have any effect on either membrane. The rat kidney isoform of the sodium–potassium pump is known to be ouabain-resistant.[18] From these experiments, it is concluded that the reduction in ATPase activity of the kidney is exclusively due to proton pump inhibition. *C. collinus* does not inhibit the Na^+^–K^+^ pump.

*Ex vivo* studies have been performed in liver and kidney cells exposed to the toxic extract. Acidification of intracellular organelles, as measured by acridine orange staining, had decreased significantly with the toxin, confirming inhibition of V-type H^+^ ATPase (vesicular proton pumps). The inhibition was comparable to known proton pump inhibitors.[20]

It is important to note here that, diphyllin, a component of the aqueous extract of *C. collinus*[21] has been shown to inhibit the V type H^+^ ATPase.[22] This is very much in accordance with our observations.

The hypercarbia in tests suggests hypoventilation. Respiratory arrest was the immediate cause of death. This could be due to respiratory muscle paralysis or central respiratory failure. It is possible to have irreversible respiratory muscle paralysis in DRTA type I due to hypokalemia.[23,24] However, in rats, although the serum potassium was lower than the corresponding values in controls, there was no frank hypokalemia. Also, in some rats, there were increased chest movements prior to the arrest [Figure 1B]. The respiratory failure observed is therefore more likely to be of central origin.

It is proposed that there is no direct cardiotoxicity due to the toxin, because the contours of the ECG complexes at the start and after cessation of respiration were not very different. The severe bradycardia and the prolonged PR interval seen terminally in the test animals can both be explained by a high vagal tone, which is expected as a result of hypoventilation or apnoea due to loss of inhibition of the vagal tone by the respiratory center.[25]

The clinical course of the poisoning in rats is similar to that in patients, in that there is alkaline urine in spite of severe acidemia and a lack of increase in potassium suggesting DRTA type I. The DRTA may worsen the clinical course, but is unlikely to be the real cause of death. Respiratory failure occurs in rats and has also been reported in human cases of *C. collinus* poisoning.[1–4] Death in rats is due to central respiratory failure, which seems to be due to a direct effect of the toxin on the respiratory center. Whether the renal and respiratory effects are due to the same or different toxic principles in the extract is being investigated currently by using isolated active principles (Diphyllin, Cleistanthins A and B) instead of the aqueous extract in the same animal model. Suitable treatment options for *C. collinus* poisoning are being addressed with the model described in this study.

The animal model of toxicity described in this study would have been better if the toxin was orally administered, which is how the patients consume the poison. However, there was aspiration with gavaging via a nasogastric tube. The intraperitoneal route was easier and hence it was preferred.

## Conclusions

Active principles of *C. collinus* inhibit proton pumps in the renal brush border, resulting in a clinical syndrome called type I DRTA in rats. This clinical picture is similar to that seen in human cases. There is no inhibition of the sodium–potassium pump activity. There is no evidence of cardiotoxicity. The rats exhibited severe respiratory acidosis and the immediate cause of death in all cases was central respiratory arrest.
